# Clinical Impact of Sarcopenia on Cholangiocarcinoma

**DOI:** 10.3390/life12060815

**Published:** 2022-05-30

**Authors:** Suk-Pyo Shin, Dong-Hee Koh

**Affiliations:** 1 Department of Internal Medicine, Chuncheon Sacred Heart Hospital, Hallym University College of Medicine, 77, Sakju-ro, Chuncheon-si 24253, Gangwon-do, Korea; pyo1029@hallym.or.kr; 2 Department of Internal Medicine, Dongtan Sacred Heart Hospital, Hallym University College of Medicine, 7, Keunjaebong-gil, Hwaseong-si 18450, Gyeonggi-do, Korea

**Keywords:** sarcopenia, cholangiocarcinoma, prognosis, survival

## Abstract

Sarcopenia is considered an important factor affecting the prognosis of cancer patients. Only complete surgical resection confers the chance of curing cholangiocarcinoma with sarcopenia. However, the prognosis is poor, even for patients who undergo surgical resection. Data from 13 trials of patients with sarcopenia and intrahepatic cholangiocarcinoma (ICC) or perihilar cholangiocarcinoma (PHC) were collected and reviewed. During all trials, sarcopenia was assessed using the psoas muscle or total skeletal muscle at the L3 level on cross-sectional images. The data showed heterogeneity among the subjects and treatment options and discrepancies in methods of measuring muscle mass and setting the cut-off level. Despite conflicting results regarding morbidity, mortality, and recurrence, sarcopenia may be associated with poor overall survival and recurrence-free survival (RFS) for ICC patients. The impact of sarcopenia on the morbidity of ICC patients remains unclear. The impact of PHC on morbidity, mortality, and RFS is also unclear. Further well-designed studies are needed to elucidate the effects of sarcopenia on ICC and PHC.

## 1. Introduction

The term “sarcopenia” was introduced by Irwin H. Rosenberg in 1989; it describes the age-related loss of muscle mass [1]. With the continued research of sarcopenia, low muscle strength and physical performance, in addition to low muscle mass, have become important diagnostic parameters for sarcopenia [2]. However, muscle strength is the primary diagnostic parameter [3]. Sarcopenia was provided with a disease code in the International Classification of Diseases, Tenth Revision, Clinical Modification in 2016 [4]; this code has matching terms in the 11th Revision [5].

Sarcopenia is associated with several human health conditions, such as cardiac disease [6,7,8], respiratory disease [9,10,11], osteoporosis [12,13,14], depression [15,16], renal function [17,18,19,20], and liver cirrhosis [21,22]. Sarcopenia in cancer patients is being actively investigated because it is an important factor in the quality of life, hospitalization time, chemotherapy-induced toxicity, postoperative complications, depressive mood, and overall survival [23,24,25,26].

Cholangiocarcinoma is a malignant tumor arising from the biliary ductal epithelium that accounts for 3% of all gastrointestinal malignancies. Among cholangiocarcinomas, 60–70% are perihilar (Klatskin tumors), 20–30% are extrahepatic, and 5–10% are intrahepatic [27]. Intrahepatic cholangiocarcinomas (ICCs) account for 10–15% of hepatic malignancies, making it the second most common primary hepatic malignancy after hepatocellular carcinoma (HCC). Because perihilar cholangiocarcinomas (PHCs) can be anatomically considered intrahepatic [28], cholangiocarcinomas can be classified as either intrahepatic or extrahepatic.

Cholangiocarcinomas are aggressive and have poor prognoses. However, cholangiocarcinomas can be cured through complete surgical resection with negative resection margins (R0). However, only 10–40% of patients can undergo surgery [29], and the 5-year survival rates for R0 intrahepatic and extrahepatic cholangiocarcinomas are 8–47% and 20–55%, respectively [30]. However, the overall 5-year survival rate is only 5–10% [29] because most patients experience liver failure, sepsis, bile duct obstruction, or cancer cachexia [31]. 

Cancer patients are vulnerable to sarcopenia. Cytokine-mediated inflammation in addition to poor nutrition and physical inactivity cause cancer patients to lose muscle mass and strength [32]. Inflammatory microenvironment in cholangiocarcinoma has an important role in the development and progression of cholangiocarcinoma, which shows aggressive features [33,34]. Thus, sarcopenia and the disease course of cholangiocarcinoma might be linked.

Recently, many studies of the relationship between cholangiocarcinoma and sarcopenia have been published. Sarcopenia influences the prognosis of surgery and chemotherapy for biliary tract cancer patients [35,36,37,38,39,40,41,42,43,44,45,46,47]. Thirteen trials of sarcopenia in cholangiocarcinoma patients, specifically those with ICC and PHC, were reviewed [35,36,37,38,39,40,41,42,43,44,45,46,47]. A summary of these trials is shown in Table 1. Studies of only distal cholangiocarcinoma patients were not included.

## 2. Assessment of Sarcopenia in Cholangiocarcinoma

Sarcopenia is associated with low muscle strength, quantity, and quality. However, these parameters are difficult to measure. Despite this difficulty, various assessment methods have been used. Muscle strength is used to assess sarcopenia, and muscle quantity or quality to confirm the diagnosis [3]. However, all the reviewed thirteen trials have assessed only radiologic image. Muscle strength and physical performance, which are important factors in accessing sarcopenia, are absent in the reviewed thirteen trials.

### 2.1. Muscle Strength

Among the parameters for sarcopenia, muscle strength has become the most important factor for its diagnosis. Muscle strength can be evaluated using the grip strength test and chair stand test [3]. Unfortunately, these tests have not been performed to analyze sarcopenia in ICC or PHC patients. 

### 2.2. Muscle Quantity

The muscle quantity can be estimated using various methods. Dual-energy X-ray absorptiometry has been widely used. Whole-body muscle mass or appendicular skeletal muscle mass (ASM) can be assessed using total body or appendicular lean soft tissue mass [48]. However, the hydration status and body thickness, as well as device and software differences, can affect the results. Magnetic resonance imaging (MRI) and computed tomography (CT) are the gold standards for estimating skeletal muscle mass (SMM) [49]. Muscle mass at the L3 level is a good representative of whole-body muscle mass [50,51]. Abdominal CT and MRI, which include L3-level images, are essential diagnostic tools for cholangiocarcinoma. Therefore, all studies of sarcopenia in ICC and PHC patients have used CT or MRI images at the L3 level to estimate skeletal muscle mass [35,36,37,38,39,40,41,42,43,44,45,46,47]. Some studies have used the total skeletal muscle area, including the rectus abdominis, erector spinae, quadratus lumborum, psoas, external and internal obliques, and trans-versus abdominis muscles, to assess sarcopenia [36,39,40,41,43,47]; however, other studies have used only the psoas muscle area [35,37,38,41,42,44,45,46]. The psoas muscle is a minor muscle; therefore, there are concerns about its ability to diagnosis sarcopenia. Some studies have shown that the psoas muscle is a good marker of sarcopenia [52]. The psoas muscle area can be assessed not only by the semi-automated method with manual outlining [35,37,38,41] but also by simple calculations using the radii of the major and minor axes of the psoas muscle cross-sections [44,46]. The latter method facilitates the easy attainment of the psoas muscle area without any specific software, although the accuracy may be compromised. 

The psoas muscle volume (PMV) can be used to diagnose sarcopenia. One study showed that the PMV was associated with postoperative complications [38].

Almost all muscle area values were normalized by height squared, regardless of the area measured. One study normalized muscle area not only by height squared but also by body surface area [45]; however, the results were insignificant. Another study measured the muscle area without normalization [44]. 

### 2.3. Muscle Density

Some previous trials reported that muscle density which reflect muscle degeneration and myosteatosis as a prognostic indicator for cancer patients [53,54,55,56,57]. Muscle density can be assessed using muscle attenuation expressed as the mean Hounsfield unit (HU) of muscle area. Some studies in the reviewed thirteen trials have measured the HU of skeletal muscle (total and/or psoas) in addition to muscle mass and analyzed the effects on survival and postoperative complications [40,41,43]. Chakedis et al. performed a sensitivity analysis and reported that muscle density was less appropriate than muscle quantity to define sarcopenia [41]. Okumura et al. reported discrepancies in muscle quantity and density when analyzing recurrence-free survival (RFS). The effect of the low skeletal muscle index on RFS was significant; however, the effect of low skeletal muscle density on RFS was not significant [40]. However, van Vugt et al. reported the opposite for survival [43]. Muscle density measurement has been investigated as a radiological method for measuring muscle quality, but has not yet been widely accepted as an accurate assessment tool for sarcopenia. 

### 2.4. Cut-Off Level

An appropriate cut-off level is important for diagnosing sarcopenia; however, no cut-off point has been universally accepted for muscle mass or muscle density. Different cut-off points have hampered efforts to achieve consistency among studies. 

The Asian Working Group for Sarcopenia and European Working Group on Sarcopenia in Older People have recommended specific cut-off points for the grip strength test, chair stand test, gait speed, timed up-and-go test, short physical performance battery, ASM, and 400 m walk test [3,58]. However, no specific cut-off value has been recommended for the cross-sectional muscle area. 

All studies of sarcopenia in cholangiocarcinoma patients used different cut-off values. Dodson et al. divided patients into four groups according to the psoas muscle index (PMI), and sarcopenia was defined as the lowest quartile [35]. Similarly, Otsuji et al. defined sarcopenia as the lowest tertile [37]. Yugawa et al. set the median level as the cut-off point [44]. Two other studies applied set points that were used for previous trials [43,45]. The other trials used statistical methods such as optimal stratification, enabling the cut-off point to reflect outcomes such as survival [36,38,39,40,41,42,46,47].

## 3. Influence of Sarcopenia on Cholangiocarcinoma

### 3.1. Morbidity

Surgical resection is the only curative option for cholangiocarcinoma patients. PHCs require extended hemi-hepatectomy with extrahepatic bile duct resection. ICCs can be treated with wedge resection, segmentectomy, major hepatectomy, extended hepatectomy, or liver transplantation, depending on tumor size and location [38,59,60]. Complications related to surgical resection include hepatic failure, bile leakage, intra-abdominal infection, surgical site infection, and sepsis [36,59]. One meta-analysis reported that the overall morbidity and severe morbidity rates after hepatectomy for PHCs were 57% and 40%, respectively [61]. 

Because major surgeries, such as hepatectomy, are stressful, sarcopenia patients are vulnerable to operative stress. Sarcopenia has been associated with higher costs and more postoperative complications [62,63].

Some trials investigating postoperative and postprocedural morbidity reported conflicting findings [35,36,37,38,39,40,41,47]. Sarcopenia was not related to morbidity after intra-arterial therapy for hepatic malignancies, including HCCs, ICCs, and metastatic lesions [35]. Furthermore, sarcopenia was not related to complications after percutaneous transhepatic biliary drainage (PTBD) for patients with PHC [47]. The other three trials did not show an association between sarcopenia and morbidity after hepatectomy [39,40,41]. Otsuji et al. reported that sarcopenia was associated with a longer hospital stay (39 days vs. 30 days; *p* < 0.001), a higher incidence of major complications with Clavien grade ≥ 3 (54% vs. 37%; *p* = 0.011), liver failure (33% vs. 16%; *p* = 0.003), and intra-abdominal abscess (29% vs. 18%; *p* = 0.040). A multivariate analysis showed that PMI values < 567 mm^2^/m^2^ for males and <395 mm^2^/m^2^ for females were risk factors for postoperative liver failure (odds ratio [OR], 2.46; 95% confidence interval [CI], 1.21–4.97; *p* = 0.012). PMI < 580 mm^2^/m^2^ for males and < 396 mm^2^/m^2^ for females were risk factors for postoperative morbidity, with a Clavien grade ≥ 3 (OR, 1.78; 95% CI, 1.07–2.98; *p* = 0.028) [37]. However, these PMIs were not consistent with the cut-off point for sarcopenia. Therefore, the association between postoperative morbidity and sarcopenia could not be confirmed by this trial. Coelen et al. showed that sarcopenia patients had increased incidences of overall complications (66.7% vs. 48.3%; *p* = 0.067), sepsis (5.2% vs. 28.6%; *p* = 0.002), and liver failure (15.5% vs. 35.7%; *p* = 0.020) in postoperative PHC patients. However, sarcopenia was not a statistically significant risk factor for overall postoperative complications (OR, 2.36; 95% CI, 0.93–5.96; *p* = 0.070) [36]. Another trial involving patients with primary liver tumors (HCC, 69.8%; ICC, 30.2%) and associated major complications reported that sarcopenia is a risk factor for postoperative morbidity (OR, 3.06; 95% CI, 1.07–8.72, *p* = 0.03); however, all 11 patients with major complications were included in the sarcopenia group [38]. In summary, some studies have reported higher postoperative morbidity rates for sarcopenia patients. However, the evidence is insufficient to establish sarcopenia as a risk factor for postoperative morbidity. Further studies are required to clearly define the impact of sarcopenia on postoperative morbidity for cholangiocarcinoma patients.

### 3.2. Overall Survival

Sarcopenia is associated with poor survival after major surgery [62] and various oncologic conditions [64,65,66,67]. One study analyzed postoperative short-term outcomes [37], whereas another 12 studies analyzed overall survival [35,36,38,39,40,41,42,43,44,45,46,47]. Two trials showed that sarcopenia was not a risk factor for poor overall survival [38,45]. During one trial, 69.8% of patients had HCC and 30.2% had ICC. Because of heterogeneity among the subjects, the results cannot be generalized to all ICC patients [38]. Another trial enrolled patients who underwent portal vein embolization, which is an uncommon procedure for cholangiocarcinoma [45].

The other ten trials indicated that sarcopenia is a predictor of poor overall survival, even when a subgroup analysis was performed [35,36,39,40,41,42,43,44,46,47]. Dodson et al. revealed that the lowest PMI showed poor survival (hazard ratio [HR], 1.84; 95% CI, 1.0–3.64; *p* = 0.004) compared to the highest PMI quartile [35]. This result was not obtained when comparing patients with and without sarcopenia. Although this trial showed the importance of muscle mass on overall survival, it did not show a predictive ability of sarcopenia for overall survival. Coelen et al. investigated PHC patients who underwent hepatectomy. The overall survival periods of the sarcopenia and non-sarcopenia groups were 22.8 and 47.5 months, respectively (*p* = 0.014). The 5-year survival rates of the sarcopenia and non-sarcopenia groups were 20.3% and 36.2%, respectively. Sarcopenia was an independent risk factor for poor overall survival (HR, 2.02; 95% CI, 1.12–3.65; *p* = 0.020) [36]. Zhou et al. also showed different survival times for the sarcopenia and non-sarcopenia groups of patients who underwent hepatectomy for hepatolithiasis-associated ICC. The median survival periods of the sarcopenia and non-sarcopenia groups were 6 and 21 months, respectively (*p* < 0.001). The ability of sarcopenia to predict poor overall survival (HR, 3.01; 95% CI, 1.65–5.51; *p* < 0.001) has also been reported [39]. 

Okumura et al. analyzed the impact of muscle mass and quality on outcomes after ICC resection. Low muscle mass (HR, 3.21; 95% CI, 1.71–6.39; *p* < 0.001) and low muscle density (HR, 3.88; 95% CI, 1.99–7.78; *p* < 0.001) were independent risk factors for poor overall survival. An analysis of the results demonstrated that patients with stage IV disease had poor survival regardless of sarcopenia. Therefore, the impact of sarcopenia on survival could only be applied to stages I–III [40]. 

Chakedis et al. analyzed the predictive value of sarcopenia on the outcomes of patients who underwent exploration for possible curative-intent resection for bile duct cancer and reported that sarcopenia, defined as decreased muscle mass, was an independent risk factor for poor overall survival (HR, 3.52; 95% CI, 1.60–7.78; *p* = 0.002). Although muscle density was not used as a parameter for sarcopenia during this study, skeletal muscle density ≤38 HU was also an independent risk factor for poor survival (HR, 2.96; 95% CI, 1.21–7.21; *p* = 0.017); however, this study included significant proportions of patients with distal cholangiocarcinoma (15%) and gall bladder carcinoma (44%) [41]. Therefore, caution is needed when applying this result to ICCs and PHCs. Yugawa et al. studied the impact of sarcopenia on ICC patients after hepatic resection. The 5-year survival rates of the sarcopenia and non-sarcopenia groups were 21.1% and 72.5%, respectively. Sarcopenia was an independent predictor of poor overall survival (HR, 2.35; 95% CI, 1.11–5.22; *p* = 0.024) [44]. Hahn et al. investigated the prognostic value of sarcopenia for ICC patients and reported that sarcopenia was an independent predictive factor for poor overall survival (HR, 1.4; 95% CI, 1.1–1.8; *p* = 0.01) of the entire cohort. However, this difference was not significant for the resected group (*p* = 0.15) and remained for the non-resected group (HR, 1.5; 95% CI, 1.0–2.1; *p* = 0.03) [42]. This report mentions the limit of sarcopenia as a criterion for the selection of operable patients. Deng et al. reported the significance of sarcopenia as a prognostic factor after curative hepatectomy for ICC patients. The absence of sarcopenia was an independent prognostic factor for overall survival (HR, 0.34; 95% CI, 0.21–0.56; *p* < 0.001) [46]. van Vugt et al. investigated the association between sarcopenia and overall survival for patients with suspected PHC, regardless of treatment modality. Low skeletal muscle density, but not a low skeletal muscle index, was associated with overall survival < 6 months (HR, 1.78; 95% CI, 1.03–3.07; *p* = 0.040), but not with survival ≥ 6 months (*p* = 0.093) [43]. 

Zhang et al. studied PHC patients who underwent PTBD. Sarcopenia was an independent predictive factor for impaired survival (HR, 3.46; 95% CI, 1.14–1.60; *p* < 0.001) [47]. In summary, almost all studies showed the predictive ability of sarcopenia for overall survival.

### 3.3. RFS

ICCs and PHCs have a dismal prognosis because of the high rate of non-resectability at diagnosis and the high recurrence rate after curative resection. A systematic review indicated that the median period until recurrence and the 3- and 5-year RFS rates after curative resection for ICC were 7–34 months, 6–47%, and 2–39%, respectively [68]. The recurrence rate after PHC resection ranges from 50% to 75% [69]. Seven trials investigated the recurrence rate or RFS. Three studies revealed that sarcopenia was not associated with recurrence [36,38,41]. Among them, one trial reported that the median disease-free survival period was not different for the sarcopenia and the non-sarcopenia groups of PHC patients (43.3 months vs. 39.8 months; *p* = 0.748) [36]. The other two trials showed patient heterogeneity [38,41]; therefore, caution is needed when interpreting these data and applying these data to ICCs and PHCs. Two studies revealed differences in overall survival but no differences in RFS regardless of sarcopenia [36,41]. Other trials have shown that sarcopenia is a risk factor for shorter RFS periods after hepatectomy for ICC [39,40,44,46].

Zhou et al. reported that sarcopenia was a risk factor for reduced RFS after hepatectomy for hepatolithiasis-associated ICC (HR, 2.06; 95% CI, 1.20–4.02; *p* = 0.011) [39]. Okumura et al. revealed that sarcopenia was an independent predictor of poor RFS (HR, 1.75; 95% CI, 1.05–2.99; *p* = 0.031); furthermore, Yugawa et al. reported that sarcopenia was a risk factor for short RFS (HR, 2.47; 95% CI, 1.06–6.01; *p* = 0.036) [40,44]. Deng et al. showed that the absence of sarcopenia was a predictor of longer RFS (HR, 0.38; 95% CI, 0.23–0.63; *p* < 0.001) [46]. Among the five studies that enrolled only ICC patients, one study did not analyze RFS [42], and the other four trials showed that sarcopenia affected RFS [39,40,44,46]. Among two studies that investigated only PHC patients [36,43], one revealed no difference [36] and the other did not investigate RFS [43]. In summary, sarcopenia might be associated with poor disease-free survival and might be an independent predictor of poor outcomes, especially with ICC. 

## 4. Discussion and Future Perspectives

Numerous studies have demonstrated the effects of sarcopenia on various oncologic conditions. Nevertheless, there has been insufficient research of sarcopenia with ICC and PHC. We reviewed the assessment of sarcopenia and the impact of sarcopenia on the outcomes of ICC and PHC patients. Only 13 studies were included in this review.

### 4.1. Limitations

This study had some limitations. The target disease and treatment modalities showed heterogeneity. Four studies enrolled patients with HCC or gall bladder cancer [35,37,38,41]. Two studies enrolled patients who underwent intra-arterial therapy [35] or PTBD [47]. Other studies enrolled patients who underwent liver transplantation [38] or portal vein embolization [45].

Cholangiocarcinoma is uncommon, and all included trials were single-center studies. Therefore, the sample size is small. Because of its rare incidence, long-term data are needed to determine the sufficient sample size. Only two studies enrolled more than 200 patients with the same disease [42,43]. One study reviewed data between 2002 and 2014 and enrolled 233 patients, and the other study analyzed data between 1997 and 2018 and enrolled 293 patients [42,43]. Many other studies retrospectively collected more than 10 years of patient data [35,36,38,39,40,41,42,43,44,45]. Cancer treatments, including surgical techniques, are developed over time. Therefore, long-term data may include heterogeneous surgical techniques, which affect consistencies in morbidity, recurrence, and survival rates.

### 4.2. Future Perspectives

Some points should be considered by future studies to clearly reveal the effects of sarcopenia on ICC and PHC patients. First, muscle strength must be investigated to assess sarcopenia. No previous study has assessed the muscle function; instead, muscle mass and muscle density have been measured. Muscle strength measurements are now standardized with the grip strength test and chair stand test [3]; therefore, future studies can use these tests with better consistency. Second, universally accepted cut-off values for variables assessing sarcopenia are necessary. The methods used to set the cut-off points for the trials were heterogeneous. The median level, lowest quartile or tertile, level based on optimal stratification, and level based on previous trials have been used. Unfortunately, there is no specific guideline setting a cut-off point for muscle mass measured by CT and MRI. Third, multicenter, prospective studies are required. The inconsistencies in the results were partially attributable to the small sample size. To create a large sample size within a short duration, patient enrollment at multiple centers is required. However, caution is needed because surgical techniques and procedural skills may be different across centers.

### 4.3. Clinical Applications

When managing patients with cholangiocarcinoma, tumor factors, such as location, size, number of intrahepatic lesions, type of tumor boundary, node and distant metastases, and tumor infiltration, and patient factors, such as age and performance status, are important. However, managing the patient according to these factors does not always produce good results. An assessment of sarcopenia may address this issue. Before tumor resection, an assessment of sarcopenia can help predict postoperative outcomes. However, there is still insufficient evidence and conflicting results regarding sarcopenia in cholangiocarcinoma patients. To date, sarcopenia alone is not a sufficient reason for excluding patients from surgery. The effect of sarcopenia on cholangiocarcinoma patients treated with chemotherapy has not yet been evaluated. Further studies should be performed to confirm sarcopenia as a prognostic factor influencing management decisions, including surgical resection and chemotherapy.

Early detection of sarcopenia in patients with cancer is important. The correction of sarcopenia might lead to better outcomes for cancer patients with sarcopenia. Kaido et al. reported that perioperative nutritional therapy consisting of branched-chain amino acids and synbiotics significantly improved the overall survival of patients with sarcopenia undergoing liver transplantation [70]. β-Hydroxy β-methylbutyrate reportedly increases lean body mass and strength of patients with advanced cancer [71,72]. Furthermore, glutamine, carnitine, creatine, fish oil, and vitamins have the potential to increase lean body mass or muscle mass [73]. Although no study of a nutritional intervention for cholangiocarcinoma patients has been performed, nutritional therapy could be a valuable treatment option.

To withstand upcoming operative stress, the functional capacity of the patient needs to be increased. Prehabilitation before surgery can improve the exercise capacity and physical function, thereby aiding postoperative recovery and preventing postoperative complications [74,75,76]. This intervention can be applied for cholangiocarcinoma patients with sarcopenia; however, the effect and exact protocol need to be further investigated.

## 5. Conclusions

Sarcopenia may affect the overall and RFS of ICC patients. However, the impact of sarcopenia on postoperative or procedural morbidity of ICC patients is unclear. Additionally, the impact of sarcopenia on patients with PHC remains unclear. Well-designed, large-scale studies are required to clarify the impact of sarcopenia on these patients.

## Figures and Tables

**Table 1:** Characteristics of the included studies.

Study/Design	Disease (*n*, %)	Treatment Option (*n*, %)	Sarcopenia Parameter	Cut-Off Level (Men/Women)	Postoperative/Procedure Morbidity (Sarcopenia vs. No Sarcopenia)	Overall Survival (Sarcopenia vs. No Sarcopenia)	Recurrence-Free Survival (Sarcopenia vs. No Sarcopenia)
Dodson et al. 2013/retrospective	Hepatic malignancy (*n* = 216) - HCC (109; 50.5%) - ICC (28; 13.0%) - CRLM (14; 6.5%) - Metastases from NELM primary (35; 16.2%) - Metastases from other primary (30; 13.9%)	Intra-arterial therapy	PMI (mm^2^/m^2^)	Lowest quartile (477; 338) mm^2^/m^2^	Overall morbidity OR, 0.89; 95% CI, 0.28–2.84; *p* = 0.84	HR = 1.84; 95% CI, 1.03–3.64; *p* = 0.04 * Lowest quartile vs. highest quartile	NR
Otsuji et al. 2015/retrospective	Bile duct cancer (218; 85%) - PHC (194; 76%) - ICC (24; 9%) Gall bladder cancer (17; 7%) Other malignant disease (6; 2%) Benign disease (15; 6%)	Resection	PMI (mm^2^/m^2^)	Lowest tertile (536; 378) mm^2^/m^2^	Major complications ^†^ 54% vs. 37%, *p* = 0.011	NR	NR
Coelen et al. 2015/retrospective	PHC (*n* = 100)	Resection	SMI (cm^2^/m^2^)	Optimal stratification (46.8; 39.1) cm^2^/m^2^	Overall morbidity OR, 2.36; 95% CI, 0.93–5.96; *p* = 0.070	HR, 2.02; 95% CI, 1.12–3.65; *p* = 0.020	43.3 months vs. 39.8 months, *p =* 0.748
Zhou et al. 2015/retrospective	Hepatolithiasis-associated ICC (*n* = 67)	Resection	SMI (cm^2^/m^2^)	Optimal stratification (43.75; 41.10) cm^2^/m^2^	Major complication ^†^ 24.2% vs. 14.7%, *p* = 0.324	HR, 3.01; 95% CI, 1.65–5.51; *p* < 0.001	HR, 2.06; 95% CI, 1.20–4.02; *p* = 0.011
Valero et al. 2015/retrospective	Primary liver tumor (*n* = 96) - HCC (67; 69.8%) - ICC (29; 30.2%)	Resection (75; 78.1%) Liver transplantation (21; 21.9%)	PMI (mm^2^/m^2^) PVI (cm^3^/m)	Optimal stratification (784.0; 642.1) mm^2^/m^2^ (34.14; 22.93) cm^3^/m	Overall morbidity OR, 3.06; 95% CI, 1.07–8.72; *p* = 0.03	HR, 1.34; 95% CI, 0.61–2.76; *p* = 0.43	5-yr RFS rate: 37.8% vs. 50.1%, *p* > 0.05
Okumura et al. 2017/retrospective	ICC (*n* = 109)	Resection	SMI (cm^2^/m^2^) SMD (HU)	Optimal stratification (52.5; 41.2) cm^2^/m^2^ (38.3; 31.0) HU	Major complication ^†^ SMI 17.4% vs. 12.5%, *p* = 0.498 SMD 18.9% vs. 12.5%, *p* = 0.360	SMI: HR, 3.21; 95% CI, 1.71–6.39; *p* < 0.001 SMD: HR, 3.88; 95% CI, 1.99–7.78; *p* < 0.001	SMI: HR, 1.75; 95% CI, 1.05–2.99; *p* = 0.031 SMD: NA
Chakedis et al. 2018/retrospective	Bile duct cancer (*n* = 117) - Distal CC (18; 15%) - PHC (27; 23%) - Gall bladder cancer (52; 44%) - ICC (20; 17%)	Exploration - Resection (78; 67%) - No resection (39; 33%)	PMI (cm^2^/m^2^)	Optimal stratification - Obesity (7.32; 5.16) cm^2^/m^2^ - No obesity (6.25; 4.21) cm^2^/m^2^	Overall morbidity 53% vs. 47%; *p* = 0.164	PMI: HR, 3.52; 95% CI, 1.60–7.78; *p* = 0.002 PMD: HR, 2.96; 95% CI, 1.21–7.21; *p* = 0.017	7.7 months vs. 12.6 months, *p* = 0.504
Yugawa et al. 2019/retrospective	ICC (*n* = 61)	Resection	PMA (cm^2^)	Median level (34.6; 18.1) cm^2^	NR	HR, 2.35; 95% CI, 1.11–5.22; *p* = 0.024	HR, 2.47; 95% CI, 1.06–6.01; *p* = 0.036
van Vugt et al. 2019/retrospective	PHC (*n* = 233) - SMI analysis: *n* = 210 - SMD analysis: *n* = 233	Resection (41; 17.6%) Open and closure (72; 30.9%) No laparotomy (120; 51.5%)	SMI (cm^2^/m^2^) SMD (HU): median level	From other trial (46.8; 39.1) cm^2^/m^2^ SMD: NR	NR	SMI: NA SMD: <6 mo HR, 1.78; 95% CI, 1.03–3.07; *p* = 0.040 ≥6 mo HR, 0.68; 95% CI, 0.44–1.07; *p* = 0.093	NR
Hahn et al. 2019/retrospective	ICC (*n* = 293)	Resection (*n* = 143) No resection (*n* = 150)	PMI (cm^2^/m^2^)	Optimal stratification (5.7, 5.1) cm^2^/m^2^	NR	HR, 1.4; 95% CI, 1.1–1.8; *p* = 0.01 Resection: HR, 1.3; 95% CI, 0.9–2.0; *p* = 0.15 No resection: HR, 1.5; 95% CI, 1.0–2.1; *p =* 0.03	NR
Deng et al. 2020/prospective cohort study	ICC (*n* = 121)	Resection	PMI (cm^2^/m^2^)	Optimal stratification (8.60, 6.04) cm^2^/m^2^	NR	HR, 0.34; 95% CI, 0.21–0.56; *p* < 0.001	HR, 0.38; 95% CI, 0.23–0.63; *p* < 0.001
Zhang et al. 2020/retrospective	PHC (*n* = 104)	PTBD	SMI (cm^2^/m^2^)	Optimal stratification (46.95; 35.14) cm^2^/m^2^	Minor: 16.4% vs. 20.9%, *p* = 0.555 Major: 19.7% vs. 11.6%, *p* = 0.275	HR, 3.46; 95% CI, 1.14–5.60; *p* < 0.001	NR
Abdelrafee et al. 2020/retrospective	Centrally located CC (*n* = 88) - PHC (79; 89.8%) - ICC extending to hilum (9; 10.2%)	Portal vein embolization - Resected (56; 63.6%) - Unresected (32; 36.4%)	PMI (mm^2^/m^2^)	From other trials (500) mm^2^/m^2^	NR	NA (*p* = 0.201)	NR

* CRLM, colorectal liver metastasis; NELM, neuroendocrine liver metastasis; NR, not reported; PMI, psoas muscle index; PVI, psoas volume index; NA, not associated; CC, cholangiocarcinoma; PTBD, percutaneous transhepatic biliary drainage. ^†^ Major complication; Clavien–Dindo grade C ≥ 3.

## Data Availability

The study did not report any data.
